# Japanese Perception of Brain Death and Implications for New Medical Technologies: Quantitative and Qualitative Social Media Analysis

**DOI:** 10.2196/54025

**Published:** 2024-09-18

**Authors:** Xanat Vargas Meza, Masanori Oikawa

**Affiliations:** 1 Institute for the Advanced Study of Human Biology Faculty of Medicine Kyoto University Kyoto Japan; 2 Department of Medical Ethics Graduate School of Medicine Tohoku University Sendai Japan

**Keywords:** brain death, Japan, social media, multidimensional analysis, Twitter, YouTube

## Abstract

**Background:**

Brain death has been used to decide whether to keep sustained care and treatment. It can facilitate tissue, organ, and body donation for several purposes, such as transplantation and medical education and research. In Japan, brain death has strict diagnostic criteria and family consent is crucial, but it has been a challenging concept for the public since its introduction, including knowledge and communication issues.

**Objective:**

We analyzed data across YouTube and Twitter in Japan to uncover actors and assess the quality of brain death communication, providing recommendations to communicate new medical technologies.

**Methods:**

Using the keyword “脳死” (brain death), we collected recent data from YouTube and Twitter, classifying the data into 5 dimensions: time, individuality (type of users), place, activity, and relations (hyperlinks). We employed a scale to evaluate brain death information quality. We divided YouTube videos into 3 groups and assessed their differences through statistical analysis. We also provided a text-based analysis of brain death–related narratives.

**Results:**

Most videos (20/61, 33%) were uploaded in 2019, while 10,892 tweets peaked between July 3 and 9, 2023, and June 12 and 18, 2023. Videos about brain death were mostly uploaded by citizens (18/61, 27%), followed by media (13/61, 20%) and unknown actors (10/61, 15%). On the other hand, most identified users in a random sample of 100 tweets were citizens (73/100, 73%), and the top 10 retweeted and liked tweets were also mostly authored by citizens (75/100, 75%). No specific information on location was uncovered. Information videos contained guides for accreditation of the National Nursing Exam and religious points of view, while misinformation videos mostly contained promotions by spirituality actors and webtoon artists. Some tweets involved heart transplantation and patient narratives. Most hyperlinks pointed to YouTube and Twitter.

**Conclusions:**

Brain death has become a common topic in everyday life, with some actors disseminating high-quality information, others disseminating no medical information, and others disseminating misinformation. Recommendations include partnering with interested actors, discussing medical information in detail, and teaching people to recognize pseudoscience.

## Introduction

### A New Concept of Death

The first concept of brain death (beyond coma) acknowledged by medical experts was present in a report by the Harvard Medical School [1]. The conceptualization shifted with advances in resuscitation and critical care; research on the physiology of consciousness; and concerns about technology, medical futility, and the ethics of end-of-life care [2]. Brain death is used to decide whether to keep sustained care and treatment, and can facilitate tissue, organ, and body donation for several purposes, such as transplantation and medical education and research. However, 55 years after its introduction, the diagnosis of brain death still varies from country to country despite efforts toward consensus [3] and calls for diagnosis based on more specific criteria [4]. Meanwhile, reactions toward brain death by the public have been mixed.

A good death can be defined as “one that is free from avoidable death and suffering for patients, families, and caregivers” [5]. Its context can vary due to age, class, culture, gender, race, and religious factors. In medical research, emerging technologies, such as DNA sequencing employed in the Rapid Autopsy Program (RAP), can also impact a good death. The RAP involves the fast-paced extraction of tissues shortly after death, where potential donors are consulted prior [6]. Japan currently has a society where more people die than are born. In 2021, there was a record of 1,439,856 deaths, with cancer being the most common cause [7,8]. Two issues are at the intersection of research for disease prevention and end-of-life care in Japan: how to integrate the views of potential donors and common people into the development of medical research techniques, such as the RAP, while acknowledging that it is difficult to discuss death and that it is not clear whether the RAP can be compatible with what Japanese people consider a good death. Understanding public acceptance of a new medical technology that has not been implemented is challenging, but we can examine brain death to compare technologies analogically.

### Contextual Aspects of Brain Death in Japan and Asia

Given the novelty of RAP procedures and the alignment of both the RAP and brain death diagnosis with Westernized models of medical practice, such arrangements can cause conflict and pain in non-Westernized contexts. From an organizational point of view, Japan can be considered as a low-power distance, collective, slightly masculine, avoidant, long-term–oriented country [9]. Japan was described as moderate in self-expression and secular, classifying it in a group of regions that includes China, Hong Kong, Macao, Mongolia, South Korea, and Taiwan, whereas there was some coincidence with Belgium, Luxembourg, Slovenia, Spain, Uruguay, and the United States in terms of self-expression [10]. For this reason, we may also refer to some literature on these countries.

In 1968, the first heart transplant in Japan from a presumed brain-dead donor was conducted by Dr Wada, and due to the lack of clear medical procedures and reporting, there were restricted transplant activities in consecutive years [11]. It is partly due to the Wada case that the Japanese diagnostic criteria for brain death are some of the strictest, if not the strictest, in the world. The first legislation recognizing brain death was passed in 1997, with amendments in the year 2010 to recognize relatives’ consent in donation decisions. The usual interpretation of the law is that brain death is human death if the patient gave prior consent to donate their organs.

Data from the Japan Organ Transplant Network indicates that 71 (93%) of 76 donation cases occurred after heart death and 5 (7%) occurred after brain death in 2000, whereas 20 years later, 68 (88%) of 77 donation cases occurred after brain death and 9 (12%) occurred after heart death [12]. Thus, although the acknowledged type of death changed, the number of donation cases remained similar. A study [13] uncovered that (1) rejection of brain death had a high association with animism, Buddhism, and Confucianism among nursing students and the public; (2) Confucianism was a more relevant factor to donate organs than brain death; and (3) the opposite was true when donating organs of kin. A survey for patients implemented through a medical magazine uncovered a positive attitude toward DNA research and banking, and the main concerns were the protection of personal information and the use of research results and their publication, whereas there was a low ability to explain brain death [14].

A survey among medical staff and the public uncovered that both groups agree that families do not want to damage the body and do not want the patient to suffer more, and an autopsy provokes suspicion and might result in accusations of medical error [15]. Yoshikawa et al [16] reported on 10 kidney transplant coordinators who participated in workshops, revealing that around two-thirds lacked knowledge on brain death before the program and that their knowledge improved after 3 months. Uncertainty about brain death criteria has decreased among the public, and Confucianism through filial piety is a barrier to organ donation [17].

In Hong Kong, a report found that most relatives do not understand what brain death is, relatives are present during brain death diagnostic tests, there is a low reluctance to accept brain death among relatives, and keeping the body intact is a major reason for donation rejection [18]. The last finding is usually a feature identified among Buddhists, but the report [18] also noted that it is a traditional Taoist belief, whereas modern Taoists believe that the body is a temporary location of the soul. A study among university students revealed that concerns about not receiving adequate medical treatment and not acknowledging brain death as human death were prevalent among some of those surveyed [19]. Further, knowledge related to organ donation (including brain death) was correlated with being in favor of organ donation in a survey among the nonmedical staff of 3 Spanish hospitals [20].

In South Korea, the satisfaction of relatives with organ donation was high due to advice received from medical experts, including the diagnosis of brain death, whereas there was lower satisfaction with the donation process and funerary arrangements, and some relief was provided through religious practices [21]. A program using educational videos reported that knowledge, attitude, and self-efficacy toward donation increased among 82 nurses [22]. The evaluation of a donation improvement program in 74 hospitals revealed that being a medical expert and having a higher medical knowledge led to an improvement in the perception of brain death [23].

In China, the consensus of brain death as human death was identified among two-thirds of medical staff, with a mean knowledge score of 8.5 out of 12, and the predictors of brain death acknowledgment included ethical acceptance, high knowledge scores, and the belief that the soul lives in the brain, while religious beliefs were unrelated [24]. A review on organ donation among Chinese Americans and Korean Americans found that Korean medical staff members were not proficient regarding brain death, more medical knowledge provided to the public did not increase postmortem donation, and Confucianism as filial piety was a barrier to donation [25].

A study in Germany argued that the rejection of the concept of brain death was mostly among Buddhist practitioners [26]. Further, a global study of Web of Science publications uncovered that the top countries publishing about brain death are the United States, Germany, Japan, France, and Spain, with Harvard University being the most active research location [27]. Moreover, the top 5 related fields were surgery (676, 35.6%), transplantation (551, 29.0%), immunology (373, 19.7%), clinical neurology (323, 17.0%), and critical care medicine (169, 8.9%), and relationships with religion were included in the discussion [27].

### Medical-Related Actors and Their Social Media Interactions

Relevant actors, their relationships, and their impacts on knowledge distribution and dissemination have been theorized in several ways. Leydesdorff and Ezkowitz [28] proposed a triple helix model involving universities, industries, and the government. Park and Stek [29] proposed a fourth helix that could include any other relevant actor, which was employed to analyze the dissemination of COVID-19 information online [30]. From a Japanese medical point of view, the role of relatives in consenting to medical procedures is crucial. This should not be seen as purely ontonomous (ie, rules established and changed by traditional cultural practices) or purely heteronomous (ie, rules established by experts and institutional bodies). Amends to the 2010 legislation would be due to the attitude of the public and the agreement of government bodies to work together with them.

Such a dynamic would correspond to the middle road of autonomy, called *kyosei* (共生; literally meaning living together) in Japan, according to Fuse [31], who argues that such a social dynamic would seek cooperation and solidarity to reach a common goal, while exposing inequalities and subordination, respecting heterogeneity, and accepting conflict. In this regard, there is some indication that associations and media outlets may be relevant communicators of brain death [32,33].

Information circulation has been impacted by social media. Studies comparing multiple platforms have uncovered diffusion patterns with various degrees of homogeneity and types of actors [34], types of content [35], information challenges [36], and information quality [37]. Japan has an internet penetration rate of 82.9%, and 74.4% of the population is active on social media [38]. Given the layer of anonymity in these spaces, people may consult, discuss, and share sensitive information about death more openly and naturally than in structured surveys or interviews [39].

### Gaps in the Reviewed Studies

There seems to be a mixed level of knowledge about brain death among medical staff and the public in the reviewed regions. Religion was operationalized in vague terms in some studies [13,21,26,27], and while some studies [13,26] reported a link between religion and acknowledgment of brain death, others [24] disproved the link. There is some indication that depending on family structure, science proficiency, and support by experts, knowledge of brain death and religion will have different levels of influence on donation consent in Japan, which in turn may affect consent for new medical research techniques such as the RAP. The role of information disseminated by mass media in brain death acknowledgment has been mentioned in few studies in Japan [17,33] but has not been thoroughly explored. Therefore, there is a need to address the issues surrounding the perception of brain death by multiple contributors, such as medical and religious actors, patients, relatives, media, and the public, in line with Japanese autonomy (*kyosei*).

### Objectives of This Study

We examined Japanese public opinion of brain death in social media through a multidimensional lens that included several actors and sociocultural factors in medical situations. We have described our methods and findings, provided the strengths and limitations of our analysis, and formulated recommendations for the communication of emerging technologies for medical research, such as the RAP, in the Japanese context. We have formulated the following objectives:

1. Identify the perception of brain death among the contributors of public Japanese discourse.
2. Identify the sociocultural aspects associated with the perception of brain death and their implications for the communication of the RAP.

## Methods

### Data Collection

Given that YouTube and Twitter (some now call it “X”) are among the top 5 most visited social networking sites in Japan [38], we collected data from these 2 social media. This allowed us to understand two types of communication formats: (1) YouTube videos that are multisensorial, last an average of 10 minutes, and are consumed by Japanese teens, adults, and elders [40], and (2) tweets that are focused on short text and used mainly by Japanese people in their twenties [41]. We used the word “脳死” (brain death) as a query.

Video data were extracted in 2023 by a researcher using YouTube Data Tools [42], which employ the YouTube application programming interface (API) through 2 modules: Video List and Video Comments. The first module retrieves a list of videos and their related information (published date, title, description, category, duration, number of views, and number of comments). Video titles were verified, and the videos were watched to discard nonmedical content, leaving a total of 61 videos from 1955. Then, identifiers (the last part of the video link) obtained through the first module were used to extract video comments with the second module.

With regard to Twitter, starting from May 2023, the number of tweets that can be collected with the free API is small and thus raises concerns about representativity. Therefore, we requested the company Tweet Binder [43] under their scheme of “historical report” to provide us with 140,000 tweets. To discard nonmedical content from the tweets, we employed the following additional keywords: “教える” (teach), “分かる” (understand), “死ぬ” (die), “臓器” (organ), “移植” (transplantation), “提供” (donation), “病院” (hospital), “ドナー” (donor), “死亡” (death), “亡くなる” (pass away), “医者” (doctor), “看護” (nursing), “マイナンバー” (my number card, an identity document), “運転免許証” (driving license card), and “健康保険証” (health insurance card), until a sample of 10,892 tweets was extracted by a researcher. We then proceeded with data classification as described in the next section.

### Data Model and Classification

**Figure 1:**
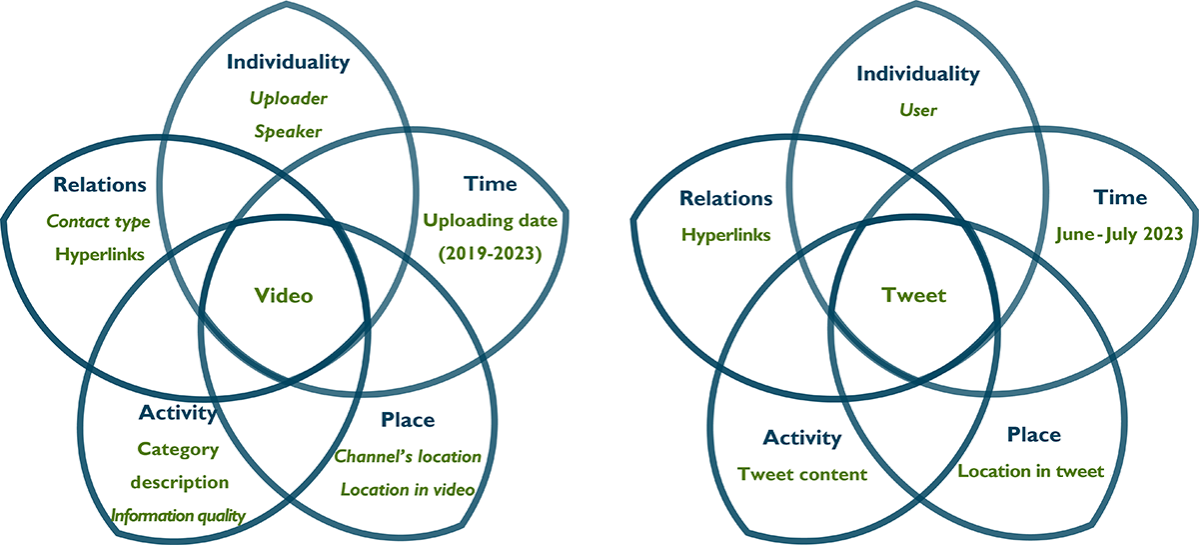
Model of social media entities to study brain death.

Considering the interaction of multiple actors and factors in line with *kyosei*, Figure 1 shows our analysis model based on [44,45], acknowledging the studied entities as YouTube videos (left) and tweets (right). Factors written in nonitalics were obtained directly from the data, whereas factors in italics were veriﬁed by an author.

The 5 main dimensions of the entities were operationalized as shown in Textbox 1.

The Brain Death Medical Information Quality (BD-MIQ) assessment is presented in Textbox 2.

**Social media entity dimensions.**

1. Time: Upload dates of YouTube videos (2019-2023) and tweet dates (June to July 2023).
2. Individuality: Uploader and speaker identified in YouTube videos. The “About” page of the corresponding YouTube channel was consulted in the case of uploaders, whereas speakers were considered as those who appear or talk, or those whose words are conveyed in the video. As for Twitter, we used a randomizer [46] to extract 100 users with average retweets and likes, and checked their profiles. The authors of the top 10 liked or retweeted tweets were also considered. These actors were classified according to information in Multimedia Appendix 1.
3. Place: The location shown in the channel’s “About” page, and the location shown in the YouTube video. For Twitter, the location in the tweet’s content was considered. Countries and regions were identified, and a place classification scheme was employed (Multimedia Appendix 2).
4. Activity: Category, description, and an information quality score (Textbox 2) based on information in [47,48] were considered for YouTube videos. For Twitter, the top frequent words in the tweet content were examined.
5. Relations: For YouTube videos, links and contact types (blog, email, social networking site, and webpage) in the descriptions were considered based on information in [30]. For Twitter, links in the tweets were considered.

**Brain Death Medical Information Quality (BD-MIQ) assessment.**

Does the video contain:

- a medically appropriate definition of brain death?
- several definitions of brain death?
- main points for diagnosis of brain death?
- additional points for diagnosis?
- caution points for diagnosis?
- exclusion criteria?
- misinformation or disinformation?

We considered misinformation as unreliable, false, deceptive, or politically charged medical information based on information in [49]. The difference between misinformation and disinformation is intention, and because it is often difficult to identify intention in media content, we decided to consider both in the same item.

### Data Analysis Techniques

We employed quantitative and qualitative techniques for data analysis. For YouTube, once the data were verified and the BD-MIQ score was calculated, the videos were classified into 3 categories: misinformation videos (any video with misinformation), no information videos (BD-MIQ score equal to zero), and information videos (all the rest). We quantified numerical results as per our research model (Figure 1), conducting statistical tests for parametric variables (uploader, speaker, place, and contact type) and nonparametric variables (published year, number of views, and number of comments) to identify significant differences between the 3 video groups. We conducted post hoc tests for significant results (threshold of *P*≤.05). We used SPSS version 29.0.10 (IBM Corp).

Text-based data (YouTube video descriptions and comments, and tweets) were processed using KH coder version 3 [50], an open software to calculate word frequency in Japanese. KH coder can summarize the most frequent words and their relationships with other words, mapping co-occurrence networks. The networks were mapped as undirected and unipartite by using the overlap coefficient (or Simpson coefficient) between 2 groups of words, where if group X is a subgroup of Y, the overlap coefficient is equal to 1 [51]. The 60 strongest co-occurrences were drawn as network edges. The Simpson coefficient generated unified networks where word groups were still identifiable.

A qualitative analysis of YouTube can be performed by labeling videos with annotations or short explanations (see [52]). We identified narratives of organ donation and transplantation that are related to brain death (Multimedia Appendix 3), compiled from a medical expert [17], a media expert [33], and a donation expert [53]. Ten YouTube videos in terms of views and comments were examined to verify the presence of narratives and the quality of information. To provide an analog analysis for Twitter, we focused on frequent words found through text analysis and revised their corresponding tweets.

### Ethical Considerations

This study was considered exempt from ethical review by the board of Kyoto University because it was conducted on social media records publicly available on the internet (Kyoto University does not provide an approval number for ethics exemption). It did not involve human data beyond measuring internet activity.

## Results

### YouTube Videos and Tweets Across Time

**Figure 2:**
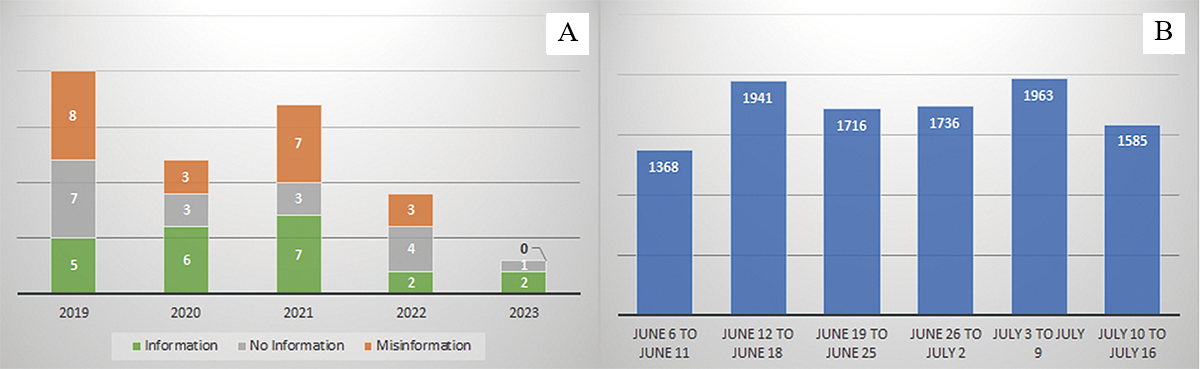
YouTube videos in Japanese about brain death uploaded between 2019 and 2023 (A) and tweets per month in 2023 (B).

Figure 2 shows YouTube videos across 5 years and tweets across June and July of 2023. Most videos (20/61, 33%) were uploaded before the COVID-19 pandemic. However, no significant (*P*=.75) differences were found between the groups. In Twitter, brain death was a constantly used term, with peaks between July 3 and 9, 2023, and June 12 and 18, 2023.

### Individuality: Actors on YouTube and Twitter

Table 1 shows the overlapping actors identified in YouTube videos, where most videos about brain death were uploaded by citizens (18/61, 27%), followed by media (13/61, 20%) and unknown actors (10/61, 15%). Statistical tests revealed a significant difference in media (*F*_2.18,8.04_=7.88; *P*=.001) between no information and misinformation videos (*P*=.001; 95% CI 0.16-0.75). This suggests that medical and patient-related actors were not prominent uploaders and that the media avoids including medical information on brain death.

In the case of speakers, doctors (24/167, 14.4%) were prominent in YouTube videos, followed by citizens (19/167, 11.4%). There was a strong difference in media speakers (*F*_3.12,8.67_=10.44; *P*<.001). The mean of media actors was different between no information and information videos (*P*=.001; 95% CI 0.17-0.78), and between no information and misinformation videos (*P*<.001; 95% CI 0.21-0.82). The results provide further confirmation that media actors did not show medical information on brain death, while other patients and their relatives rarely appeared in videos with medical information on brain death.

With regard to Twitter, the assessment of a sample of 100 users with 1 retweet revealed that 73 (73%) identified as citizens, whereas among 100 users with 3 likes, 75 (75%) were identified as citizens. Tables 2 and 3 summarize users with the top likes and retweets, with their usernames anonymized in the case of individuals. There was mostly an unclear position on brain death reflected in their tweets, with some of the top users being nurses, patients, and patient relatives. Those who acknowledged brain death included a nurse and patient, 3 citizens, and the Kyodo News media company.

**Table 1:** Actors in YouTube videos according to the Brain Death Medical Information Quality (BD-MIQ) assessment.

Actor	IU^a^ (n=26), n	NU^b^ (n=19), n	MU^c^ (n=20), n	TU^d^ (n=66), n	IS^e^ (n=55), n	NS^f^ (n=54), n	MS^g^ (n=58), n	TS^h^ (n=167), n
Doctor	2	0	0	2	8	8	8	24
Medical student	0	0	0	0	1	1	0	2
Nurse	2	0	0	2	6	4	3	13
Other medical staff	2	1	0	3	3	1	1	5
Donor	0	0	0	0	5	0	4	9
Recipient	0	0	0	0	2	0	1	3
Donor relative	0	0	0	0	5	2	2	9
Recipient relative	0	0	0	0	1	0	1	2
Other patient	0	1	0	1	1	8	6	15
Other patient relative	0	0	1	1	1	7	8	16
Association	2	2	0	4	0	1	0	1
Citizen	4	2	12	18	7	3	9	19
Government	1	0	0	1	2	4	4	10
Media	3	9^i^	1^i^	13	3^i^	11^i^	2^i^	16
Religion	6	0	2	8	5	0	2	7
Education	2	0	1	3	3	2	2	7
Unknown	2	4	4	10	2	2	5	9

^a^IU: information uploaders.

^b^NU: no information uploaders.

^c^MU: misinformation uploaders.

^d^TU: total uploaders.

^e^IS: information speakers.

^f^NS: no information speakers.

^g^MS: misinformation speakers.

^h^TS: total speakers.

^i^Statistically significant (*P*<.05) difference.

**Table 2:** Top Twitter users according to likes.

Username^a^	Likes, n	Type	Acknowledgment of brain death
@aaaaa	2019	Patient	Unclear
@bbbbb	998	Patient relative	Unclear
@ccccc	495	Unknown	Unclear
@ddddd	255	Citizen	Yes
@eeeee	238	Citizen	Unclear
@fffff	225	Nurse, patient	Yes
@ggggg	168	Citizen	Yes
@hhhh	162	Citizen	Unclear
@iiiii	160	Citizen	Yes
@jjjjj	159	Nurse	Unclear

^a^Usernames are anonymized in the case of individuals.

**Table 3:** Top Twitter users according to retweets.

Username^a^	Retweets, n	Type	Acknowledgment of brain death
@aaaaa	689	Patient	Unclear
@bbbbb	486	Patient relative	Unclear
@ccccc	203	Unknown	Unclear
@kkkkk	66	Citizen	No
@eeeee	56	Citizen	Unclear
@mmmmm	40	Citizen	Yes
@lllll	35	Citizen	No
@jjjjj	33	Nurse	Unclear
@iiiii	20	Citizen	Yes
@kyodo_official	18	Media	Yes

^a^Usernames are anonymized in the case of individuals.

### Locations of YouTube Videos and Tweets

Table 4 displays the types of overlapping places and Japanese locations identified in YouTube videos. Most places were closed, and locations in Japan were mostly unclear at the regional level. Further, the videos showed or discussed locations elsewhere in the world, including the United States (8/61, 13%); China (4/61, 7%); South Korea (3/61, 5%); Cambodia, Czech Republic, France, and North Korea (2/61, 3%); and Australia, Brazil, Spain, Taiwan, and the United Kingdom (1/61, 2%). No specific information on location was uncovered for Twitter.

**Table 4:** Places and locations in YouTube videos according to the Brain Death Medical Information Quality (BD-MIQ) assessment.

Place		Information (n=69), n	No information (n=50), n	Misinformation (n=57), n	Total (n=176), n
Hospital		7	10	11	28
Medical office		3	0	3	6
Educational institution		1	1	2	4
Home		6	6	10	22
Religious building		4	0	1	5
Other closed space		10	9	9	28
Nature		6	3	1	10
Open space		5	4	5	14
Unknown		7	0	1	8
**Location in Japan**					
	Chubu	2	2	0	4
	Chugoku	1	1	1	3
	Kansai	1	1	0	2
	Kanto	0	3	2	5
	Kyushu	2	0	1	3
	Other	14	10	10	34

### Activities in YouTube Videos and Tweets

The most frequent categories chosen by YouTube video uploaders were People & Blogs (22/61, 36%), News & Politics (13/61, 21%), Entertainment (9/61, 15%), and Education (8/61, 13%). The average BD-MIQ score of the 61 videos was 0.77, with 24 (39%) including the definition of brain death, 22 (36%) including additional definitions of death, 14 (23%) discussing the main points of diagnosis, 9 (15%) discussing additional points of diagnosis, and none discussing caution points of diagnosis or exclusion criteria. Additionally, of the 61 videos, 22 (36%) were classified as information, 18 (30%) were classified as no information, and 21 (34%) were classified as misinformation.

Although there were no significant differences in terms of views (*P*=.22) or comments (*P*=.32), a review of the top 10 viewed videos uncovered that 6 were classified as misinformation, 2 as information, and 2 as no information, including 5 webtoons (digital comics), 2 news videos, 1 video about rumors, 1 video about fake news, and 1 video about a Japanese drama. Regarding the 10 most commented videos, 5 were classified as misinformation, 4 as no information, and 1 as information, including 4 webtoons, 2 video blogs discussing beliefs of brain death, 2 videos about rumors, 1 video about fake news, and 1 news video. None of these top videos were uploaded by medical actors, and only 1 video blog was uploaded by a patient, while the news video and drama video contained medical information on brain death.

**Figure 3:**
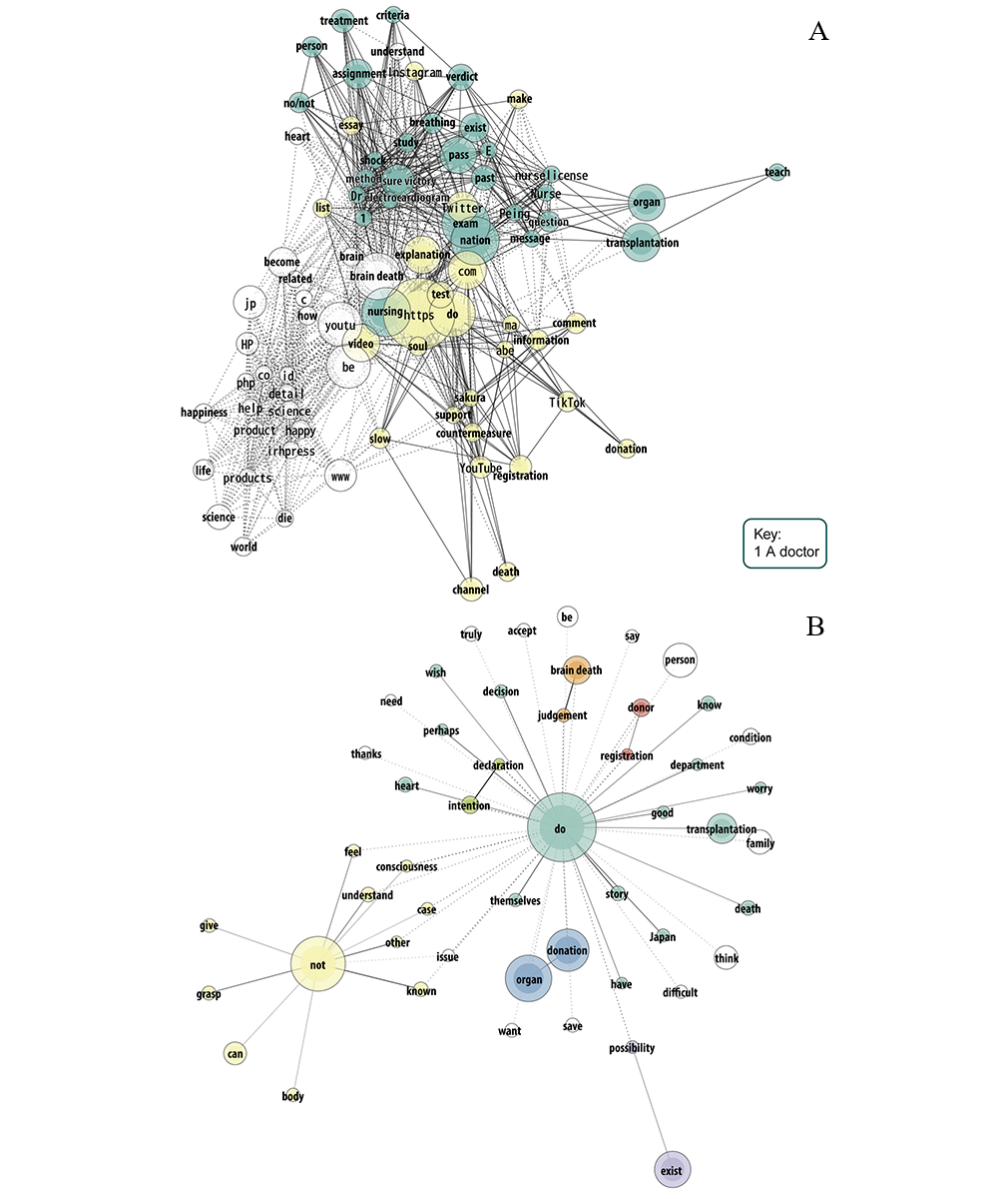
Descriptions (A; word frequency 4 to 60; tie strength 0.6 to 1) and comments (B; word frequency 35 to 1000; tie strength 0.6 to 0.96) of 22 YouTube information videos in Japanese about brain death.

**Figure 4:**
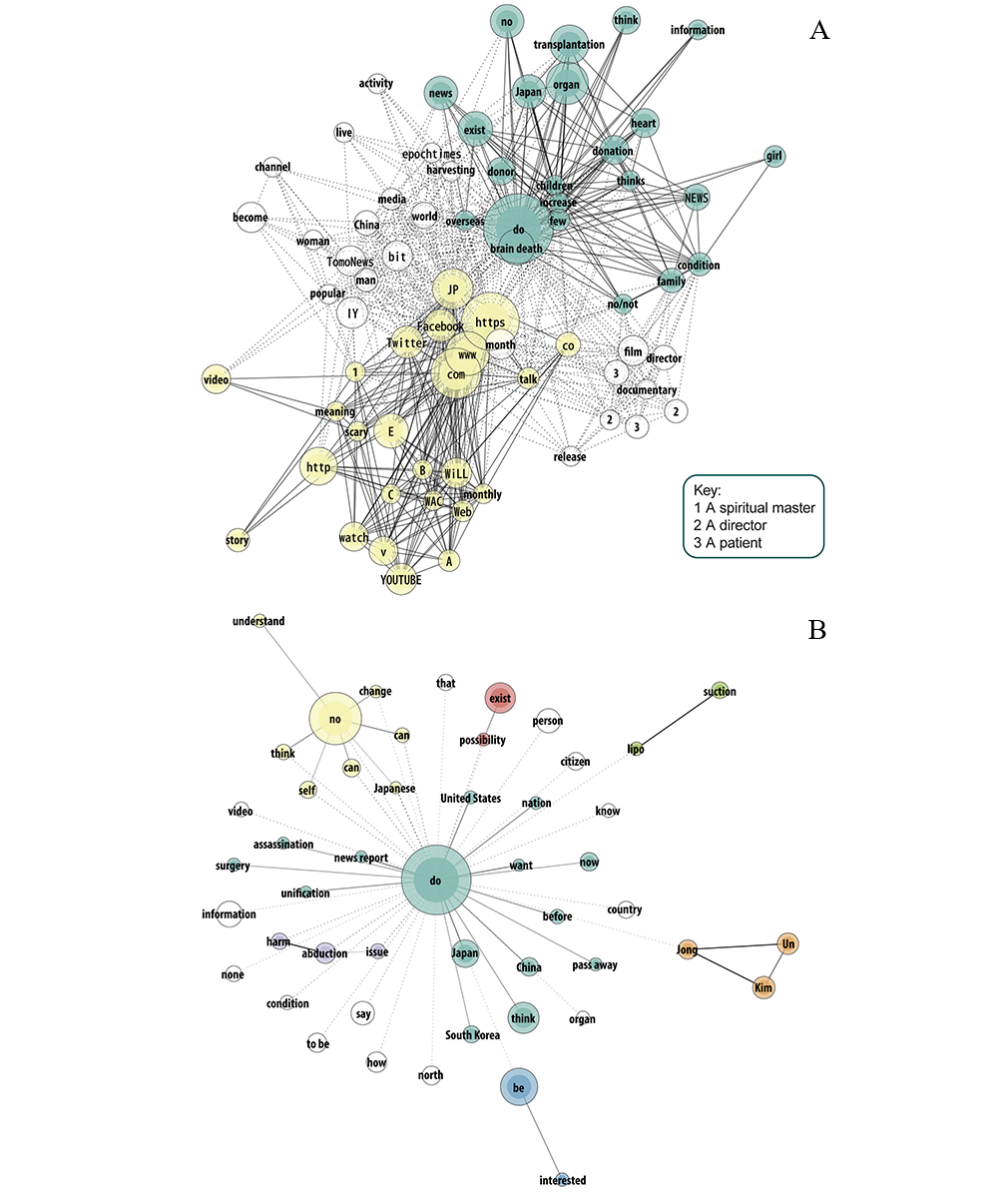
Descriptions (A; word frequency 5 to 60; tie strength 0.6 to 1) and comments (B; word frequency 50 to 1500; tie strength 0.6 to 0.9) of 18 YouTube no information videos in Japanese about brain death.

**Figure 5:**
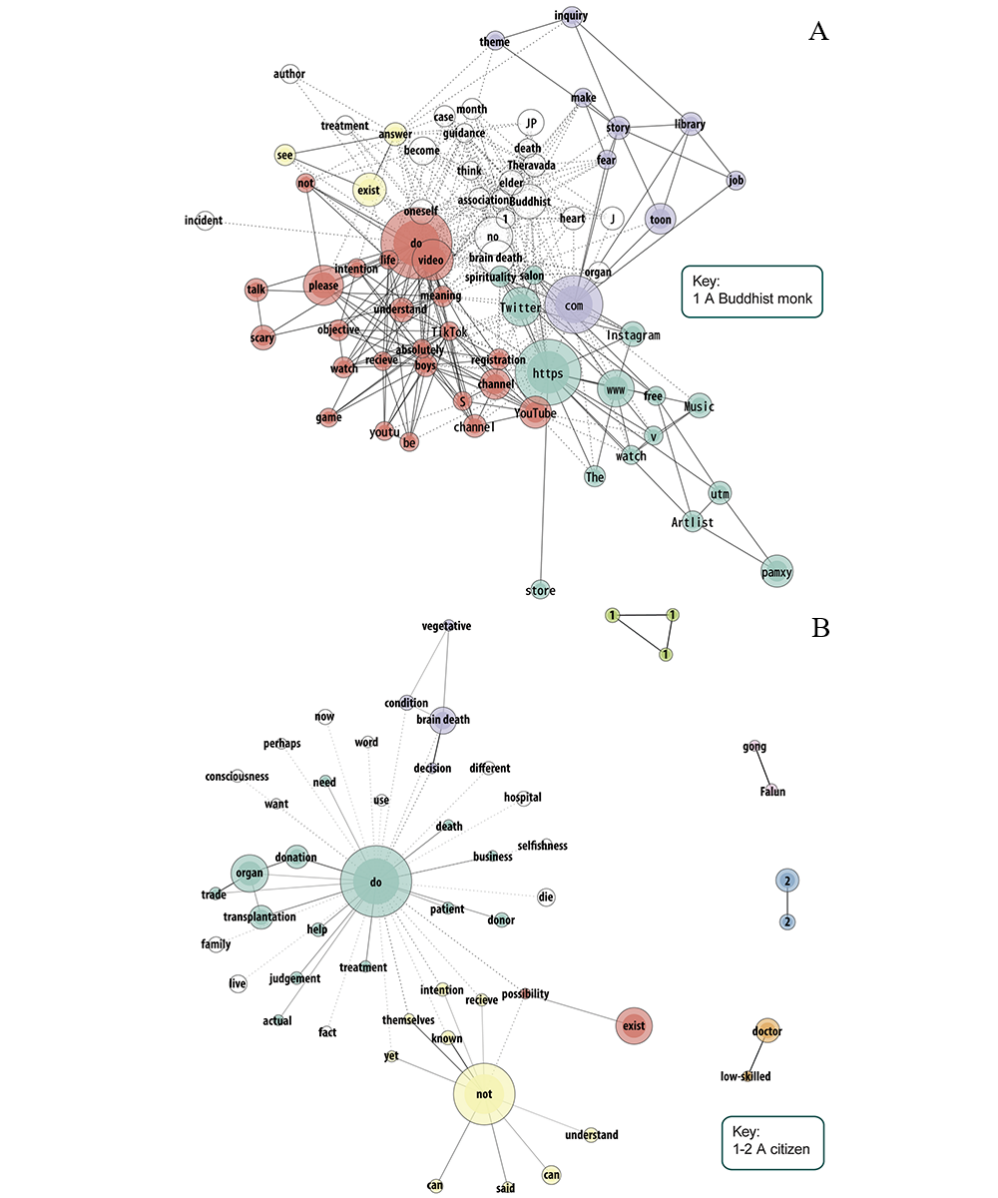
Descriptions (A; word frequency 4 to 60; tie strength 0.6 to 1) and comments (B; word frequency 50 to 2000; tie strength 0.6 to 1) of 21 YouTube misinformation videos in Japanese about brain death.

Figures 3-5 show the most frequent words employed in video descriptions and their comments across groups. The node size reflects word frequency, and a thicker line indicates a stronger tie strength. Nodes without color do not correspond to a particular group. In the case of descriptions of information videos (Figure 3), a cluster (in green) corresponded to content for accreditation of the National Nursing Exam uploaded by doctors, nurses, education-related actors, and unknown actors, which included brain death diagnosis criteria. Another cluster (in yellow) overlapped test content uploaded by nurses, with other content discussing brain death from a medical and spiritual point of view, including 3 videos by Buddhist monks from the Jodo and Tendai sects. Among other frequent words, promotion content uploaded by new religion actors discussed brain death from a medical and spiritual point of view.

As for comments on information videos, a large group (in green) included the terms “good,” “decision,” and “themselves,” and this was linked to a group that included the phrase “organ donation,” a group with the phrase “declaration (of) intention,” and another group with the phrase “donor registration,” suggesting that what happens after brain death is a decision taken by the individual and that it has to be clearly expressed and documented. Given the words “heart” and “transplantation,” it seems that this is one of the most commonly acknowledged procedures after brain death. Another cluster (in yellow) corresponded to negative opinions of brain death, including the terms “not,” “understand,” “known,” “grasp,” “consciousness,” “feel,” and “other,” which may be interpreted as failures to understand brain death and that people outside the patient cannot make decisions based on this lack of knowledge. Other words included “accept,” “family,” “difficult,” “need,” “save,” and “want,” highlighting the role of relatives in determining death and some altruism. Most comments were inclined to be active (“do”) instead of being negative (“not”).

Regarding the description of no information videos (Figure 4), a cluster (in green) corresponded to news reports on organ transplantation and donation information videos in Japan. Several of these videos were related to pediatric patients and their relatives. Another group (in yellow) included descriptions of 2 videos about a spiritual healer who allegedly cured a patient from a near brain death condition. Other words included documentaries and news regarding a patient near brain death, and there were 2 reports by foreign news media channels (“epochtimes” and “TomoNews”), with one discussing illegal organ harvesting in China and the other discussing birth by a brain-dead woman in Czech Republic.

Comments made in these videos included a large word group (in green) comparing news in China, Japan, South Korea, and the United States. Several reacted to a rumor initiated by American media companies CNN and NBC [54] and carried by others about the brain death of North Korean leader Kim Jong Un due to liposuction, using the word “assassination.” Another group (in yellow) included the terms “no,” “change,” “understand,” “Japanese,” and “self,” implying that Japanese people do not understand brain death. Another cluster (“abduction,” “harm,” and “issue”) corresponded to illegal organ harvesting news in China. There was an international dimension in the comments, and they did not display a clear acknowledgment of brain death. The term “brain death” itself was used 129 times but was not connected to any of the main clusters and hence was not included in the figure.

Regarding the description of misinformation videos (Figure 5), a cluster (in green) consisted of promotions by spirituality actors and artists, and it was linked to other groups (in purple and red) about scary webtoon channels, suggesting that spirituality actors and webtoon artists are highly involved in the generation of misinformation regarding brain death. Among other words, a representative of Theravada Buddhism discussed brain death in a video, implying that the person may still be alive. “Heart” was also among the most frequently used words, providing more evidence for heart transplantation as a common topic.

As for comments, a word group (in green) included terms, such as “organ,” “donation,” “transplantation,” “judgment,” “business,” and “trade,” implying that brain death diagnosis is made for profit. Another cluster (in yellow) included the words “not,” “understand,” “Japanese,” and “yet,” suggesting that Japanese people do not understand brain death. The word group that included the term “brain death” (in purple) also included “vegetative,” suggesting that the commenters did not distinguish between these 2 conditions. Other clusters disconnected from the largest ones (grass green and blue) pertained to 2 people (one who spammed the comments section and another who received aggressive comments after apparently taking a webtoon seriously). Another group (in pink) was based on a new religion from China, which was related with an organ harvesting video. Moreover, another group (in orange) discussed “low-skilled doctors.” Among other terms, we can mention “family” and “hospital.” In general, the comments appeared to be suspicious and negative toward brain death.

**Figure 6:**
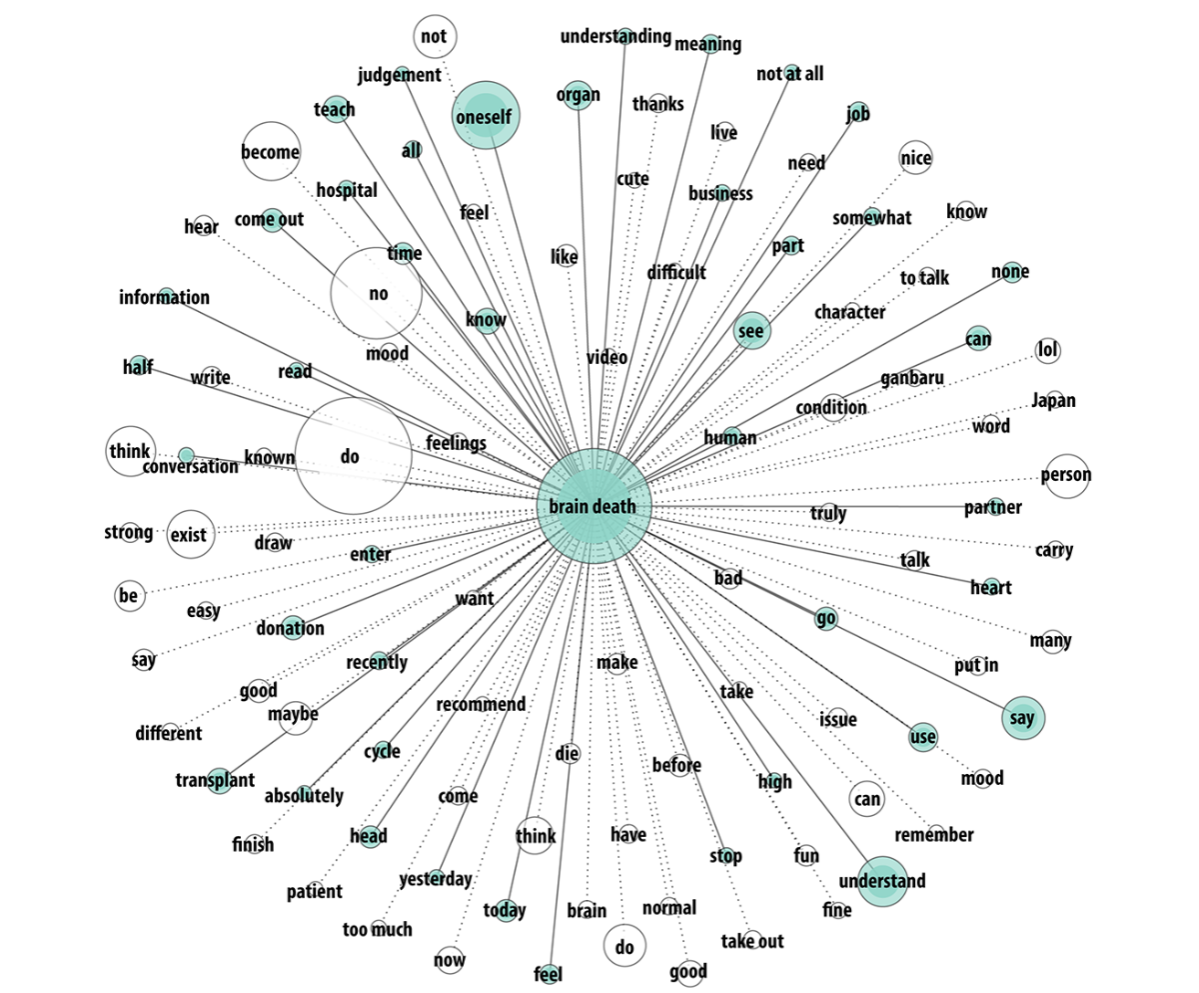
The most frequent words in 10,892 Japanese tweets about brain death (word frequency 150 to 9000; tie strength 0.6 to 1).

On Twitter, there was only 1 cluster connected through the keyword “brain death” (Figure 6), which suggests that the topics were diversified. Brain death was mostly understood, although the term “Japan” was separated from the cluster. The word “heart” was included in this cluster, providing evidence that heart-related interventions are a common topic on Twitter too. The term “ganbaru” was also among the most frequent words. Illegal organ harvesting news from China was also present in the tweets, but they were not prominent and thus were not displayed in the figure.

### Relations: Beyond YouTube and Twitter

Of the 61 YouTube videos, 28 (46%) included social media links, 24 (39%) included webpage links, 5 (8%) included an email, and 1 (2%) included a blog link. The only variable with a median over zero was webpage links, and it was not significantly different across video groups (*P*=.30). Figures 4 to 6 provide hints regarding popular links employed by actors related to brain death. In YouTube information videos, webpages, YouTube, Twitter, and some presence of Instagram and TikTok could be noted. In no information videos, webpages were mentioned, followed by similar frequencies of Facebook, Twitter, and YouTube. Lastly, in misinformation videos, most links were those related to art websites and online stores, followed by Twitter and YouTube, and there was a lesser presence of Instagram and TikTok. This suggests that Twitter and YouTube are the most consistent social media employed to communicate about brain death, whereas there is more confirmation of art-related actors spreading misinformation.

As for Twitter, a review of the tweets uncovered that links were usually directed to other tweets and to comics and pictures, confirming the role of artists in the spread of information related to brain death on this social media platform.

### Brain Death Narratives on YouTube and Twitter

We found that most narratives in YouTube videos were unclear in their acknowledgment of brain death. We also found additional narratives to those in Multimedia Appendix 3. These narratives were as follows:

- The potential donor looks alive: the body is warm, the heart continues to beat, etc. Identified in 7 (11%) of 61 videos, with 4 (7%) classified as misinformation and 3 (5%) classified as no information. Discussed by artists, a media channel, an educational channel, a patient, and an unknown source.
- Mechanistic view of life: the body works like a computer, and its parts can be used as spare parts. Identified in 4 (7%) information videos and discussed by Jodo and Tendai Buddhist monks acknowledging brain death and spiritualist actors against brain death acknowledgment.
- Organ harvesting and profit: brain death involves economic exploitation. Identified in 4 (7%) videos, with 3 (5%) classified as misinformation and 1 (2%) classified as information. Of the 4 videos, 2 were uploaded by unknown sources, 1 by a medical staff member, and 1 by a media channel.
- Brain-dead people can feel pain: identified in a video blog by a citizen.
- Brain death is equal to a vegetative state: identified in a webtoon uploaded by an artist.

The following narratives acknowledged brain death and were in favor of acting in consequence:

- Continuity of life: identified in 5 (8%) of 61 videos, with 3 (5%) classified as misinformation, 1 (2%) classified as no information, and 1 (2%) classified as information. Mentioned by donor relatives.
- Ganbaru: identified in 4 (7%) videos, with 2 (3%) classified as information, 1 (2%) classified as no information, and 1 (2%) classified as misinformation. Mentioned by donor and patient relatives.
- Gift of life: identified in 1 (2%) video, which was classified as information and uploaded by an association.
- Make the intention for donation clear: involved discussing the topic with relatives and using additional means besides a donor card to express donation intention. It was identified in 2 (3%) videos, with 1 (2%) classified as information and 1 (2%) classified as misinformation. The videos were uploaded by associations.
- Political consequences of brain death: the person was considered as unable to perform their political duties. Identified in 2 (3%) videos classified as no information and uploaded by a news agency and a citizen.
- Giving happiness to others: identified in 1 (2%) video classified as information and mentioned by donor relatives.

Lastly, the following set of narratives did not acknowledge brain death:

- Brain death is not human death: mostly argued that heart death is human death. Identified in 8 (13%) of 61 videos, with 4 (7%) classified as information and 4 (7%) classified as misinformation. Discussed by citizens and educational and religious actors.
- People can recover from brain death: identified in 4 (7%) videos classified as misinformation, with 3 (5%) being webtoons uploaded by citizens.
- Individualized spirituality: death should not be determined by doctors, and medical decisions should be made based on religious beliefs. Identified in 2 (3%) videos classified as misinformation. The argument was combined with Gotai Manzoku and linked to Buddhism by new religion actors.

A review of the top tweets in terms of likes and retweets uncovered that those who did not acknowledge brain death argued that people who experience it can feel pain and that they can recover. Additionally, there was some mistrust toward the government and associations tied to organ donation.

## Discussion

### Summary of the Main Findings

On YouTube, medical actors were not active uploaders, while on Twitter, a few medical actors, notably nurses, communicated about brain death. This finding is similar to the diffusion of cancer information on Twitter in Japan [37]. A relevant finding of this study was the high knowledge of some religious actors regarding brain death, including Jodo, Tendai Buddhism, and new religion representatives. The implication is that such actors, particularly Jodo and Tendai Buddhism representatives, may be able to discuss new medical interventions, such as the RAP with common people, as they discuss death openly and Buddhist rituals are common procedures after death in Japan. There are few examples of the effectiveness of this approach in the realm of organ transplantation [55].

A large majority of brain death–related videos and tweets were identified in hospitals and other closed environments, away from other specific end-of-life care environments and communities, such as those in hospices and homes. Although most Japanese people die in hospitals, other end-of-life care environments seem to be increasing [56], and it would be advisable to examine brain death in such contexts.

There were distinctive reactions in YouTube comments depending on the BD-MIQ. While comments made in information videos seemed inclined to acknowledge brain death, comments in no information videos were confused, and commenter impressions were negative in misinformation videos. Major issues with the acceptance of organ donation (whether it is for profit, medical negligence, or overseas transplantation) were prominent in misinformation videos but were mostly absent in videos with high BD-MIQ scores. The qualitative review of tweets pointed to similar issues, prompting caution in the acknowledgment of brain death.

### Social Media Actors and Their Roles in the Communication of Brain Death

The presence of many medical actors as speakers in videos was due to their image or footage being used in news reports, webtoons, etc. In webtoons, medical actors were often portrayed as looking for profit, morally corrupt, or lowly skilled. Patients and their relatives were largely inactive users, and their image or footage was used in news reports and webtoons. Some patients and relatives unrelated to organ donation were active in the discussion of brain death, although their knowledge level was low. There was a mismatch between interest in the diffusion of medical topics and expertise in the topics, which should prompt medical actors interested in new research procedures, such as the RAP, to seek patients and relatives displaying overlapping interests to create collaboration partnerships.

Japanese medical actors tend to consider that the media portrays brain death in a sensationalized way, violating the privacy of those involved [17]. However, the reviewed content by Japanese established media largely avoided sensationalization and most did not contain medical information about brain death in both fictionalized (eg, drama) and nonfictionalized (eg, news reports) media. In contrast, we found sensationalization and misinformation in the content released by Japanese webtoon channels and foreign media. Communication guidelines regarding organ donation recommend avoiding medical details and focusing on the positive impacts of donation in humanized stories [57]. It seems that a large portion of Japanese established media has adopted these recommendations, while a considerable portion of new media does not follow them.

Regarding religious actors, Tendai Shu Buddhism, an esoteric school, was founded in the early Heian period (806 DC) by the monk Saicho, while Jodo Shinshu (Pure Land) is the most practiced Buddhism in Japan, founded by the monk Shinran who wrote extensively on spiritual matters during the 13th century [58,59]. Their Buddhist representatives provided nuanced discussions about brain death, being inclined to accept it as human death and to act in consequence. On the other hand, the representative of Theravada Buddhism, founded in India and based on meditation practices, voiced an opinion including information and misinformation.

Based on our findings, it seems that some new religious movements do not recognize brain death as human death. Further, there is a global trend toward the reduction of organized religion, with spiritualism being a type of diffused religion where individuals choose what to believe and practice according to their needs [59]. This may prompt commercialization, hyperindividualization, and superficiality of religious beliefs and practices, which seem to be present in videos by spiritualist actors. It is noted that speakers related to a new religion linked their arguments against brain death to Gotai manzoku, the preservation of the body according to old Taoist, Confucianist, and Buddhist beliefs, which was not an argument employed by Buddhist representatives. Therefore, a person who apparently displays a belief from a majoritarian religion might be a practitioner of a new religion or spirituality that opposes contemporary medical practices and would not be open to procedures such as the RAP.

Long [60] discussed religious scripts followed when a person is close to death in Japan, placing Buddhist scrips as a distinctive branch with Indian origins that mixed with Chinese and Korean cultures before arriving in Japan, whereas Taoism and Confucianism (the root of Gotai manzoku) are in a branch of their own. Given our findings, we consider that we should not place all Buddhist scripts in the same category, as some are being used to refuse brain death and others are being used to accept it. Moreover, despite their Chinese origin, Taoist and Confucianist arguments also should be treated separately, as new Taoist ideas acknowledge brain death in Asian regions [18].

Moreover, we argue that a new type of religious script (individualized spirituality) from new religions and spiritualist actors should be considered. In this script, death is not determined by medical actors, emphasizing personally held values in opposition to medical procedures. Moreover, outdated ideas of older religions against the acknowledgment of brain death are recycled. Whenever such scripts overlap with new Japanese Buddhist scripts, there may be an opportunity to establish a dialogue between those in favor and those against new medical procedures such as the RAP.

### Quality Levels of Information and Commenter Behaviors

Most viewed and commented videos about brain death were created by citizens (including a few artists) who mixed medical information with misinformation. Misinformation is thus sophisticated and hard to identify for common people. Although such content is often considered as entertainment and not to be taken seriously, as per our qualitative review of misinformation comments, its potential harm should not be underestimated. Moreover, there were a few videos by media companies and individuals employing rumors. Although we considered them as no information, if the audience has enough overlap with beliefs expressed in the videos, there is a risk of taking them seriously [61]. The audience that consumes misinformation and no information is larger than the one that consumes information about brain death partly due to the tendency of ranking algorithms to prioritize content that receives negative and strong reactions, and monetized content [62,63].

The accessibility of information by the public should be assessed based on internet formats and user interaction. van Dijk [64] divided digital skills into technology-based skills and digital literacy, where the ﬁrst skill focuses on the operation of digital devices, and the latter focuses on creating, ﬁnding, processing, and communicating information. There are some gaps in digital accessibility in Japan [65], which implies that rural regions and elders might have more difficulty in accessing recent scientific information. There is also evidence of low health literacy on Twitter in Japan [37], which seems to be on par with the English-speaking public. Therefore, medical actors interested in implementing new research technologies, such as the RAP, must be aware of misinformation, rumors, and digital divide aspects to address the medical information needs of research participants.

Scientific experts often employ specialized language and diffuse their knowledge in formats that target other experts in their field, such as highly priced books and colloquiums. Such communication formats are often outside the scope of interest of the public, while misinformation employs formats that are appropriate for entertainment, attractive, rapidly accessible, and easy to consume. A relevant but uncommon example of such a format among information videos was a 59-second video explaining brain death by a medical association, which obtained high engagement.

### Recommendations for RAP Communication in Japan

Based on our findings, we considered communication strategies of the RAP based on *kyosei* and prebunking. Prebunking is a process similar to vaccination and consists of the following 2 steps: forewarning and pre-emptive refutation of misinformation [66]. First, there should be an evaluation of the community where the RAP is potentially going to be implemented, considering: (1) age, (2) altruism level, (3) interest in science, (4) digital (information) literacy, (5) technology-based skills, (6) educational level, (7) personal medical diagnosis and diagnosis among relatives, (8) religious affinity, (9) trust in medical experts, and (10) trust in governments.

By considering these variables, medical experts can identify people who may be interested in the implementation of the RAP in their communities and work with them to not only prioritize the required information but also identify potential exposure to misinformation and unclear medical information. Further, Fagan [67] proposed 11 criteria to identify pseudoscience. Among these criteria, we can provide the following pseudoscience scripts to be prebunked:

1. Dogged adherence to outdated theoretical models: such as in Gotai manzoku versus updated Japanese Buddhist scripts. Can be prebunked together with religious actors [55].
2. Disparaging academia: such as in the negative portrayals of medical actors in webtoons.
3. Appeal to academic authority: such as in the use of medical actors and settings by foreign news reports and webtoons.
4. Huge claims: such as in frequently used keywords (eg, “scary talk”) in videos.
5. Selective or distorted presentation: such as in the use of medical actors and settings in foreign news reports and webtoons.
6. Vague definitions: although it is considered unadvisable by communication experts, our text analysis across YouTube video groups and the low science literacy on Twitter suggest that in the case of Japanese citizens, it is necessary to provide detailed information to prompt positive reactions toward new medical technologies.
7. Superficiality, sloppiness, and grossness of comparison: mostly found in videos by patients, relatives, and common citizens with low brain death knowledge. This can be mitigated if medical experts educate and work together with these interested actors.

There seems to exist an underdeveloped use of social media as communication ties with potential donors, relatives, and the public. Twitter seems to be used more for promotion than for interaction with users in real time, when it could be employed to quickly correct medical misinformation [68]. Medical experts can also partner with artists who are interested in science and technology to diffuse the RAP. Although few, there were some high-quality information webtoons uploaded by unidentified actors. Another aspect in line with *kyousei* to promote the RAP would be to provide psychological support for a good death that includes religious actors, such as in Buddhist-based therapy [58,69].

Based on YouTube categories and text analysis, although new medical procedures are the realm of science and technology, tying them to people and daily life topics would facilitate their diffusion among citizens. It seems that the best scripts to communicate about the RAP are those that place the continuity of life and empathy toward the patient (Ganbaru) upfront, tying them to potential positive outcomes.

### Strengths and Limitations

We have provided a thorough description of content across 2 platforms that focus on different types of formats, targeting different representative audiences. We successfully identified multiple opinions by diverse actors in the Japanese social media landscape, which informed recommendations for the communication of the RAP that might be useful for other new medical technologies.

Regarding limitations, the keyword “脳死” (brain death) was mostly related to being tired and thoughtless, and was tied to video game scenarios. It was thus challenging to identify medical content on Twitter and YouTube. To understand more about medical information scenarios in Japanese social media, it is recommended to test other medical-related keywords and combinations of them. Further, this study did not address the potential impact of cultural diversity on brain death perception, as we could only identify a few Japanese regions in the videos. Therefore, future studies should consider more data and methods to measure cultural diversity and should consider other contexts beyond Japan.

### Conclusion

We analyzed the perception of brain death in Japanese social media, providing recommendations for the communication of the RAP. Based on our objectives, our conclusions are presented in Textbox 3.

**Conclusions.**

1. Regarding time:
  - Brain death has become part of the vocabulary of common people, being used constantly in a large range of scenarios.
2. Regarding individuality:
  - Patients and their relatives mostly appeared in misinformation videos.
  - Citizens uploaded most videos about brain death, and most of them included medical misinformation.
  - Religious uploaders included medical information on brain death. While Japanese Buddhist representatives acknowledged brain death, new religion and spiritualist actors did not acknowledge it.
  - Japanese media mostly omitted medical information on brain death.
3. Regarding place:
  - Most environments were closed and connected to hospitals.
4. Regarding activity:
  - Information on brain death was diffused by medical actors and a few unidentified actors, eliciting positive reactions.
  - No information elicited confusion.
  - Misinformation was disseminated by citizens and a few foreign media outlets, eliciting negative reactions toward brain death.
5. Regarding relations:
  - YouTube and Twitter are the most suitable social media to communicate about brain death.
6. Regarding recommendations:
  - Medical actors (nurses in particular) can partner with interested patients, relatives, citizens, and religious actors to communicate about the RAP.
  - Include environments besides hospitals to engage people in their daily lives.
  - Medical information should be discussed.
  - An emphasis on brain death diagnosis procedures should be made.
  - Misinformation and rumors should be addressed by teaching people to recognize pseudoscience and other common scripts related to new medical technologies.

## Appendix

Multimedia Appendix 1 Actor classification.

Multimedia Appendix 2 Place classification.

Multimedia Appendix 3 Narratives related to brain death.

## Abbreviations

API: application programming interface
BD-MIQ: Brain Death Medical Information Quality
RAP: Rapid Autopsy Program

## Data Availability

Anonymized data sets are available at [70].

## References

[1] Committee of the Harvard Medical School to Examine the Definition of Brain Death (1968). A definition of irreversible coma. Report of the Ad Hoc Committee of the Harvard Medical School to Examine the Definition of Brain Death. JAMA.

[2] De Georgia MA (2014). History of brain death as death: 1968 to the present. J Crit Care.

[3] Greer DM, Shemie SD, Lewis A, Torrance S, Varelas P, Goldenberg FD, Bernat JL, Souter M, Topcuoglu MA, Alexandrov AW, Baldisseri M, Bleck T, Citerio G, Dawson R, Hoppe A, Jacobe S, Manara A, Nakagawa TA, Pope TM, Silvester W, Thomson D, Al Rahma H, Badenes R, Baker AJ, Cerny V, Chang C, Chang TR, Gnedovskaya E, Han M, Honeybul S, Jimenez E, Kuroda Y, Liu G, Mallick UK, Marquevich V, Mejia-Mantilla J, Piradov M, Quayyum S, Shrestha GS, Su Y, Timmons SD, Teitelbaum J, Videtta W, Zirpe K, Sung G (2020). Determination of brain death/death by neurologic criteria: The World Brain Death Project. JAMA.

[4] Omelianchuk A, Bernat J, Caplan A, Greer D, Lazaridis C, Lewis A, Pope T, Ross LF, Magnus D (2022). Revise the Uniform Determination of Death Act to align the law with practice through neurorespiratory criteria. Neurology.

[5] Gustafson DH (2007). A good death. J Med Internet Res.

[6] Hooper JE, Williamson AK (2019). Autopsy in the 21st Century: Best Practices and Future Directions.

[7] Summary of vital statistics. Ministry of Health, Labour and Welfare.

[8] Trends in leading causes of death. Ministry of Health, Labour and Welfare.

[9] Country comparison tool. Hofstede Insights.

[10] (2023). The Inglehart-Welzel World Cultural Map. World Value Survey.

[11] Watanabe K (2013). Japanese and Westerners' views on life and death in the brain death/organ transplant debate. Annual Report on Death and Life.

[12] Kadota M (2022). Status and challenges of organ donation in Japan.

[13] Saita N, Sasaki M, Okate K, Sato S, Azuma R (2006). People's perception on organ transplantation and human death - using religious factor scale for view of death and life. Japanese Society of Nursing Proceedings: Comprehensive Nursing.

[14] Kobayashi E, Satoh N (2009). Public involvement in pharmacogenomics research: a national survey on public attitudes towards pharmacogenomics research and the willingness to donate DNA samples to a DNA bank in Japan. Cell Tissue Bank.

[15] Maeda S, Kamishiraki E, Starkey J, Ikeda N (2013). Why are autopsy rates low in Japan? Views of ordinary citizens and doctors in the case of unexpected patient death and medical error. J Healthc Risk Manag.

[16] Yoshikawa M, Yoshinaga K, Imamura Y, Hayashi T, Osako T, Takahashi K, Kaneko M, Fujisawa M, Kamidono S (2016). Transplant procurement management model training: Marked improvement in the mindset of in-hospital procurement coordinators at Hyogo Prefecture, Japan. Transplant Proc.

[17] Akabayashi A (2020). Bioethics Across the Globe: Rebirthing Bioethics.

[18] Lee WC The change of attitudes towards organ donation in Hong Kong. University of Hong Kong Scholars Hub.

[19] Wong SH, Chow AYM (2021). Preliminary development of a postmortem bodily integrity concerns scale among the university students in Hong Kong. Omega (Westport).

[20] Ríos A, López-Navas A, Ayala-García MA, Sebastián MJ, Abdo-Cuza A, Alán J, Martínez-Alarcón L, Ramírez-Barba EJ, Muñoz-Jiménez G, Palacios G, Suárez-López J, Castellanos R, González-Yebra B, Martínez-Navarro MÁ, Díaz-Chávez E, Nieto A, Ramírez P, Parrilla P (2013). Attitudes of non-medical staff in hospitals in Spain, Mexico, Cuba and Costa Rica towards organ donation. Nefrologia.

[21] Kim H, Yoo Y, Cho O (2014). Satisfaction with the organ donation process of brain dead donors' families in Korea. Transplant Proc.

[22] Oh HS, Park MA (2019). The effect of an educational intervention regarding human tissue donation on nurses' knowledge, attitude, and education-related self-efficacy. J Korean Acad Soc Nurs Educ.

[23] Heo SJ, Ju YH, Noh EJ, Kim KM, Son YK, Jung SW, Kang HJ, Lee JR, Cho WH, Ha J (2021). A study on the performance of the Donation Improvement Program in Korea. Korean J Transplant.

[24] Yang Q, Fan Y, Cheng Q, Li X, Khoshnood K, Miller G (2015). Acceptance in Theory but not Practice – Chinese Medical Providers’ Perception of Brain Death. Neuroethics.

[25] Li MT, Hillyer GC, Husain SA, Mohan S (2019). Cultural barriers to organ donation among Chinese and Korean individuals in the United States: a systematic review. Transpl Int.

[26] Alhawari Y, Verhoff MA, Ackermann H, Parzeller M (2020). Religious denomination influencing attitudes towards brain death, organ transplantation and autopsy-a survey among people of different religions. Int J Legal Med.

[27] Doğan G, Kayır S (2020). Global scientific outputs of brain death publications and evaluation according to the religions of countries. J Relig Health.

[28] Leydesdorff L, Etzkowitz H (1996). Emergence of a Triple Helix of university-industry-government relations. Science and Public Policy.

[29] Park HW, Stek P (2022). Measuring helix interactions in the context of economic development and public policies: From triple to quadruple and n-tuple helix vs. n-tuple and quadruple helix to triads. Triple Helix.

[30] Zhu YP, Park HW (2021). Development of a COVID-19 web information transmission structure based on a quadruple helix model: Webometric network approach using Bing. J Med Internet Res.

[31] Fuse M, Kohtari A, Salleh A, Escobar A, Demaria F, Acosta A (2019). Kyosei. Pluriverse: A Post-Development Dictionary.

[32] Nawa N, Ishida H, Suginobe H, Katsuragi S, Baden H, Takahashi K, Narita J, Kogaki S, Ozono K (2018). Analysis of public discourse on heart transplantation in Japan using social network service data. Am J Transplant.

[33] Huguet Cañamero E (2022). The normalization of organ donation discourse in the press.

[34] Park SJ, Lim YS, Park HW (2015). Comparing Twitter and YouTube networks in information diffusion: The case of the “Occupy Wall Street” movement. Technological Forecasting and Social Change.

[35] Struck JP, Siegel F, Kramer MW, Tsaur I, Heidenreich A, Haferkamp A, Merseburger AS, Salem J, Borgmann H (2018). Substantial utilization of Facebook, Twitter, YouTube, and Instagram in the prostate cancer community. World J Urol.

[36] Saleh I, Mba C (2023). Utilization of YouTube and Twitter as source of information for research by medical students at Usmanu Danfodiyo University, Sokoto. Journal of Health Information and Librarianship.

[37] Kureyama N, Terada M, Kusudo M, Nozawa K, Wanifuchi-Endo Y, Fujita T, Asano T, Kato A, Mori M, Horisawa N, Toyama T (2023). Fact-checking cancer information on social media in Japan: Retrospective study using Twitter. JMIR Form Res.

[38] Kemp S Digital 2023: Japan. DataReportal.

[39] Murphy AK, Jerolmack C, Smith D (2021). Ethnography, data transparency, and the information age. Annu Rev Sociol.

[40] Share of people who use YouTube in Japan in fiscal year 2022, by age group. Statista.

[41] Share of people who use Twitter in Japan in fiscal year 2022, by age group. Statista.

[42] YouTube Data Tools. Digital Methods.

[43] Tweet Binder.

[44] Vargas Meza X, Park HW (2023). Information circulation among Spanish-speaking and Caribbean communities related to COVID-19: Social media-based multidimensional analysis. J Med Internet Res.

[45] Vargas Meza X, Oikawa M (2024). Japanese perception of organ donation and implications for new medical technologies: Quantitative and qualitative social media analyses. JMIR Form Res.

[46] Number List Randomizer. Number Generator.

[47] Takeuchi K, Iinuma K, Ogawa Y, Kamoshita S, Sakai H, Satoh H, Shiogai T, Shimazaki S, Sugimoto H, Takeshita H, Tanaka H, Nihei K, Nukui H, Matsumoto S, Miyasaka K, Momma K, Watanabe Y (2002). Report on the criteria for the determination of brain death in children. JMAJ.

[48] Araki T, Yokota H, Fuse A (2016). Brain death in pediatric patients in Japan: Diagnosis and unresolved issues. Neurol Med Chir (Tokyo).

[49] Altay S, Berriche M, Acerbi A (2023). Misinformation on misinformation: Conceptual and methodological challenges. Social Media + Society.

[50] Higuchi K (2016). A two-step approach to quantitative content analysis: KH coder tutorial using Anne of Green Gables (Part I). Ritsumeikan Social Science Review.

[51] Vijaymeena MK, Kavitha K (2016). A survey on similarity measures in text mining. Machine Learning and Applications: An International Journal (MLAIJ).

[52] Park H, Lim Y (2020). Do North Korean social media show signs of change?: An examination of a YouTube channel using qualitative tagging and social network analysis. Journal of Contemporary Eastern Asia.

[53] Yasuoka MK (2015). Organ donation in Japan: A medical anthropological study.

[54] Tillison T CNN, NBC float report Kim Jong Un’s brain dead after recent surgery – social media’s odd response. Bizpac Review.

[55] Hayashima O (2022). 生命操作と仏教 -選択される生命と生き方の選択 (Life Manipulation and Buddhism: Choosing Life and the Way of Life).

[56] Yang L, Sakamoto N, Marui E (2006). A study of home deaths in Japan from 1951 to 2002. BMC Palliat Care.

[57] Dominguez-Gil B (2022). Cómo se ha conseguido la aceptación social de la donación de órganos en España.

[58] Gisho S (2019). Dharma-based person-centered approach in Japan. Psychological Counseling and Psychotherapy.

[59] Roth C, Rambelli F (2019). Essays in Vagueness: Aspects of Diffused Religiosity in Japan. Spirits and Animism in Contemporary Japan: The Invisible Empire.

[60] Long SO (2005). Final Days: Japanese Culture and Choice at the End of Life.

[61] Junker A (2016). Live organ harvesting in China: Falun Gong and unsettled rumor. Am J Cult Sociol.

[62] Son J, Lee HK, Jin S, Lee J (2019). Content features of tweets for effective communication during disasters: A media synchronicity theory perspective. International Journal of Information Management.

[63] Caplan R, Gillespie T (2020). Tiered governance and demonetization: The shifting terms of labor and compensation in the platform economy. Social Media + Society.

[64] van Dijk JA, Rössler P (2017). Digital Divide: Impact of Access. The International Encyclopedia of Media Effects.

[65] Nishida T, Pick JB, Sarkar A (2014). Japan's prefectural digital divide: A multivariate and spatial analysis. Telecommunications Policy.

[66] Basol M, Roozenbeek J, Berriche M, Uenal F, McClanahan WP, Linden SVD (2021). Towards psychological herd immunity: Cross-cultural evidence for two prebunking interventions against COVID-19 misinformation. Big Data & Society.

[67] Fagan GG (2006). Archaeological Fantasies: How Pseudoarchaeology Misrepresents the Past and Misleads the Public.

[68] Lim D, Toriumi F, Yoshida M (2021). Do you trust experts on Twitter?: Successful correction of COVID-19-related misinformation.

[69] Kato H (2016). The relationship between the psychology of religion and Buddhist psychology. Jpn Psychol Res.

[70] Vargas Meza X, Oikawa M (2024). Anonymised social media datasets related to brain death in Japan. Zenodo.

