# Supplementary material for: Japanese Perception of Brain Death and Implications for New Medical Technologies: Quantitative and Qualitative Social Media Analysis
Source: JMIR Form Res. 2024 Sep 18;8:e54025. doi: 10.2196/54025 (PMC11447421; doi:10.2196/54025)
Supplement: Multimedia Appendix 1 [file formative_v8i1e54025_app1.pdf]

Multimedia Appendix 1. Actor classification.

Actor Type	Subtype	Description
Medical Staff	Doctor	Surgeons, doctors, therapists
	Medical student	Medicine and nursing students
	Nurse	Nursing professionals
	Other medical staff	Secretaries, donor coordinators, hospital personnel
Patients and Relatives	Donor	Organ/tissue donors
	Recipient	Organ/tissue recipients
	Donor relative	Organ/tissue donor relatives
	Recipient relative	Organ/tissue recipient relatives
	Other patient	
	Other patient relative	
Others	Association	Organizations for dignified death, disease prevention, organ donation, etc.
	Citizen	Common people
	Government	Government officials, judges, police
	Media	Journalists and media companies
	Religion	Religious heads, preachers, devotees
	Education	Non-medical researchers, educators, students
	Unknown	Actors that could not be classified in other categories.
