# Supplementary material for: Japanese Perception of Brain Death and Implications for New Medical Technologies: Quantitative and Qualitative Social Media Analysis
Source: JMIR Form Res. 2024 Sep 18;8:e54025. doi: 10.2196/54025 (PMC11447421; doi:10.2196/54025)
Supplement: Multimedia Appendix 3 [file formative_v8i1e54025_app3.pdf]

Multimedia Appendix 3. Narratives related to brain death.

Actor	Narrative	Description
Heart transplant doctor	Harvesting life[a]	
	Harvesting love[a]	
Patient	Altruism[b]	Consists of prioritizing family in health decisions.
	お任せ(Omakase)[b]	To leave health decisions to their family.
Patient relatives	頑張る (Ganbaru)[ab]	The patient is doing/did their best during their struggle with illness.
	五体満足(Gotai manzoku)[ab]	The wish to preserve the patient's body intact.
Japan Organ Transplantation Network	Connect life[c]	Through donation, the lives of several people are connected.
	Continuity of life[ac]	The patient continues to live in someone else.
	Gift of life[a]	
	Rebirth of life[a]	Also called "renewable life", donation gives new life to donor and recipient.
	Relay of life[ac]	The life of the patient passes on to others.
	Giving life[c]	A summary of all the other narratives by this actor.

[a] Based on [1]; [b] based on [2]; [c] based on [3]

References

1. Yasuoka MK. *Organ donation in Japan: A medical anthropological study*. Lexington Books, 2015.
2. Akabayashi A. *Bioethics Across the Globe: Rebirthing Bioethics*, Springer Open, 2020: 13-26.
3. Huguet Cañamero E. The normalization of Organ Donation discourse in the press. *Organ donation in Japan and Spain - Possibilities of the Spanish model*. Tokyo Japan, 2022 January 25.
